# Unintended perturbation of protein function using GFP nanobodies in human cells

**DOI:** 10.1242/jcs.234955

**Published:** 2019-11-01

**Authors:** Cansu Küey, Gabrielle Larocque, Nicholas I. Clarke, Stephen J. Royle

**Affiliations:** Centre for Mechanochemical Cell Biology, Warwick Medical School, University of Warwick, Gibbet Hill Road, Coventry CV4 7AL, UK

**Keywords:** Clathrin-mediated endocytosis, Dynamin, Nanobody, GFP-binding protein, Knocksideways

## Abstract

Tagging a protein of interest with GFP using genome editing is a popular approach to study protein function in cell and developmental biology. To avoid re-engineering cell lines or organisms in order to introduce additional tags, functionalized nanobodies that bind GFP can be used to extend the functionality of the GFP tag. We developed functionalized nanobodies, which we termed ‘dongles’, that could add, for example, an FKBP tag to a GFP-tagged protein of interest, enabling knocksideways experiments in GFP knock-in cell lines. The power of knocksideways is that it allows investigators to rapidly switch the protein from an active to an inactive state. We show that dongles allow for effective knocksideways of GFP-tagged proteins in genome-edited human cells. However, we discovered that nanobody binding to dynamin-2–GFP caused inhibition of dynamin function prior to knocksideways. The function of GFP-tagged tumor protein D54 (TPD54, also known as TPD52L2) in anterograde traffic was also perturbed by dongles. While these issues potentially limit the application of dongles, we discuss strategies for their deployment as cell biological tools.

This article has an associated First Person interview with the first author of the paper.

## Abstract (precis)

**Highlighted Article:** Functionalized GFP-binding nanobodies (dongles) can extend the use of GFP-tagged proteins. Here, dongles that allow knocksideways are demonstrated, but they are limited by perturbation of the target protein caused by dongle binding alone.

## INTRODUCTION

Fluorescent proteins revolutionized cell biology. The green fluorescent protein (GFP) or its relatives can be attached to virtually any protein of interest and allow the direct visualization of that protein by light microscopy or flow cytometry (Wang and Hazelrigg, 1994). Whole genome GFP-tagging projects have been completed in yeast (Huh et al., 2003), plants (Tian et al., 2004), bacteria (Kitagawa et al., 2005) and fly (Nagarkar-Jaiswal et al., 2015). The advent of genome engineering, particularly via CRISPR/Cas9, has allowed the creation of GFP knock-in mammalian cell lines in labs around the world (Jinek et al., 2013), with centralized efforts to systematically tag genes in human induced pluripotent stem cells (Roberts et al., 2017). While these resources are incredibly useful, additional tags would further enhance our ability to probe protein function in single cells.

Of particular interest is the ability to rapidly modulate protein function. Inducible methods such as relocation (Haruki et al., 2008; Robinson et al., 2010) and degradation (Nishimura et al., 2009) allow investigators to study the effect of inactivating a protein of interest in live cells. For example, we have used the knocksideways method to study protein function at distinct stages of mitosis, without perturbing interphase function (Cheeseman et al., 2013). Here, a protein of interest has an FKBP tag that allows inducible binding to a mitochondrially targeted protein containing an FRB tag (MitoTrap) via the heterodimerization of FKBP and FRB by rapamycin (Robinson et al., 2010). The power of these methods lies in the comparison of the active and inactive states of the protein of interest.

The development of camelid nanobodies that bind GFP have been very useful as affinity purification tools (Rothbauer et al., 2008). Since these nanobodies can be readily expressed in cells, it is possible to use them as ‘dongles’ to extend the functionality of GFP by attaching a new protein domain to the GFP-tagged protein of interest via fusion with the nanobody. This approach has been exploited to degrade proteins of interest (Caussinus et al., 2011; Kanner et al., 2017; Daniel et al., 2018; Yamaguchi et al., 2019), to introduce additional tags (Rothbauer et al., 2008; Ariotti et al., 2015; Derivery et al., 2017; Zhao et al., 2019), or to constitutively relocalize GFP-tagged proteins (Schornack et al., 2009; Derivery et al., 2015). Recently a suite of functionalized nanobodies to GFP or RFP were generated, enabling recoloring, inactivation, ectopic recruitment and calcium sensing (Prole and Taylor, 2019). The dongle approach holds much promise because it is flexible and saves investigators from re-engineering knock-in cell lines to introduce additional tags.

Some time ago, we developed dongles to allow knocksideways experiments in GFP knock-in cell lines. The approach certainly works and we demonstrate this using two different genome-edited human cell lines. However, we discovered during the course of development that nanobody binding to dynamin-2–GFP causes inhibition of dynamin function, prior to any induced inactivation. Since the purpose of knocksideways is to compare active and inactive states, the dongles could not be used in this way. The aim of this paper is to alert other labs to the possibility that nanobodies against GFP can perturb the function of the target GFP-tagged protein. We discuss what strategies investigators might pursue as alternatives and outline possible applications of dongles despite this limitation.

## RESULTS

### Testing fluorescent protein selectivity of dongles in cells

**Fig. 1.**
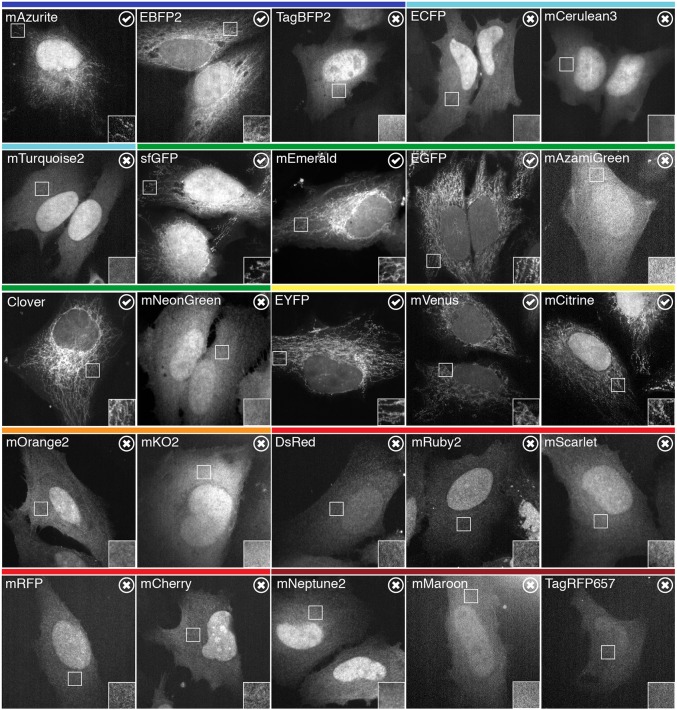
**Selectivity of dongles for fluorescent proteins.** Representative images of HeLa cells expressing DongleTrap (pMito–GBPen) and the indicated fluorescent protein. Binding to DongleTrap results in mitochondrial localization of the fluorescent protein and is indicated by a tick (check mark). Fluorescent proteins that do not bind to the DongleTrap remain cytoplasmic and are indicated by a cross. Insets show a 2× zoom of the indicated region of interest. Colored bars above indicate the approximate emission of the fluorescent proteins tested.

Most experimental applications of dongles would involve two different fluorescent proteins, one as a target for the dongle and a second as an experimental readout. We therefore wanted to assess the fluorescent protein selectivity of the GFP nanobody in cells. To do this, we used a visual screening method in HeLa cells by expressing a GFP nanobody (GFP-binding protein enhancer, GBPen) that was constitutively attached to the mitochondria (DongleTrap, see Materials and Methods) along with a suite of twenty-five different fluorescent proteins. Affinity of the fluorescent protein for the DongleTrap resulted in a steady-state relocation to the mitochondria, while lack of interaction meant that the fluorescent protein remained cytoplasmic (Fig. 1). We observed relocation for mAzurite, EBFP2, sfGFP, mEmerald, EGFP, Clover, EYFP, mVenus and mCitrine. The following fluorescent proteins remained cytoplasmic in all cells examined: TagBFP2, ECFP, mCerulean3, mTurquoise2, mAzamiGreen, mNeonGreen, mOrange2, mKO2, DsRed, mRuby2, mScarlet, mRFP, mCherry, mNeptune2, mMaroon and TagRFP657. All of the fluorescent proteins that DongleTrap binds are derivatives of avGFP (GFP from *Aequorea victoria*), while it did not bind proteins from other lineages, e.g. dsRed, eqFP578, and LanYFP (Lambert, 2019). The GBPen has further specificity besides lineage, since DongleTrap did not bind other avGFP descendants ECFP, mCerulean3 and mTurquoise2 (Kubala et al., 2010). These experiments demonstrated which tags could be manipulated by dongles in cells (e.g. GFP), but also which fluorescent proteins can be used simultaneously with these tools, without interference (e.g. mCherry).

### Dongles can be used to extend the function of GFP

**Fig. 2.**
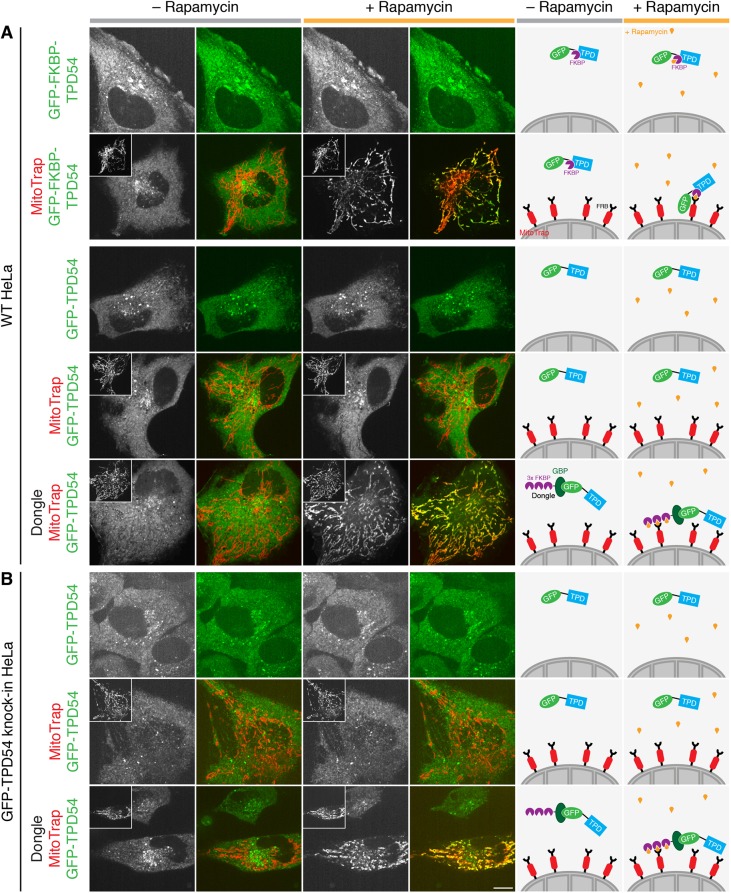
**Dongles allow for knocksideways of GFP-tagged proteins that have no FKBP tag.** Representative confocal images of live cells taken before (gray bar) or after (orange bar) addition of 200 nM rapamycin. (A) GFP–FKBP–TPD54 or GFP–TPD54 were expressed in wild-type (WT) HeLa cells, along with MitoTrap (pMito-mCherry-FRB) alone or together with the dongle as indicated. If MitoTrap (red in merge) was co-expressed, the red channel is shown in the inset at half size. (B) In GFP–TPD54 knock-in HeLa cells, MitoTrap or MitoTrap+dongle were expressed as indicated. Scale bar: 10 µm. Schematic diagrams to the right illustrate the experimental conditions and the respective result.

Knocksideways is a useful tool to rapidly inactivate proteins by sequestering them onto mitochondria using heterodimerization of FKBP and FRB domains (Robinson et al., 2010). Typically, the FKBP domain is fused to the protein of interest (usually along with GFP for visualization) and the FRB domain is part of MitoTrap (Fig. 2). To demonstrate the usual application of this method, we rerouted the membrane trafficking protein tumor protein D54 (TPD54, also known as TPD52L2) to mitochondria (for detailed analysis of TPD54 rerouting see Larocque et al., 2019). To do this, we expressed GFP–FKBP–TPD54 in HeLa cells either alone or together with MitoTrap. Application of 200 nM rapamycin caused the relocation of GFP–FKBP–TPD54 to mitochondria in seconds, only when MitoTrap was present (Fig. 2A).

We wanted to use knocksideways on proteins that have a GFP tag, but no FKBP. To enable this we designed a dongle comprising three copies of FKBP fused to the N-terminus of GBPen, which can be co-expressed in cells along with MitoTrap (see Materials and Methods). When expressed transiently in cells along with GFP–TPD54, application of rapamycin (200 nM) caused GFP–TPD54 to become rapidly rerouted to mitochondria (Fig. 2A). Mitochondrial rerouting was dependent on the presence of the dongle, since no rerouting was seen in rapamycin-treated cells expressing GFP–TPD54 and MitoTrap. The effect was indistinguishable from rerouting of GFP–FKBP–TPD54 to MitoTrap in response to rapamycin (Fig. 2A).

Given these encouraging results, we next tested whether dongles could be used to reroute endogenous proteins, tagged with GFP, to the mitochondria. To do this, we expressed the dongle and MitoTrap in cells where endogenous TPD54 was tagged with GFP (Larocque et al., 2019). We found that GFP–TPD54 was rerouted when the dongle and MitoTrap were present and rapamycin was added (Fig. 2B). Knocksideways was qualitatively similar to GFP–FKBP–TPD54 or GFP–TPD54 and dongle, expressed with MitoTrap in wild-type HeLa cells (Fig. 2). These experiments indicate that the dongles can be used to extend the function of GFP and to permit knocksideways experiments in GFP knock-in cell lines without an FKBP tag. We termed this method ‘dongle-knocksideways’.

### Knocksideways of dynamin-2 in gene-edited human cells

**Fig. 3.**
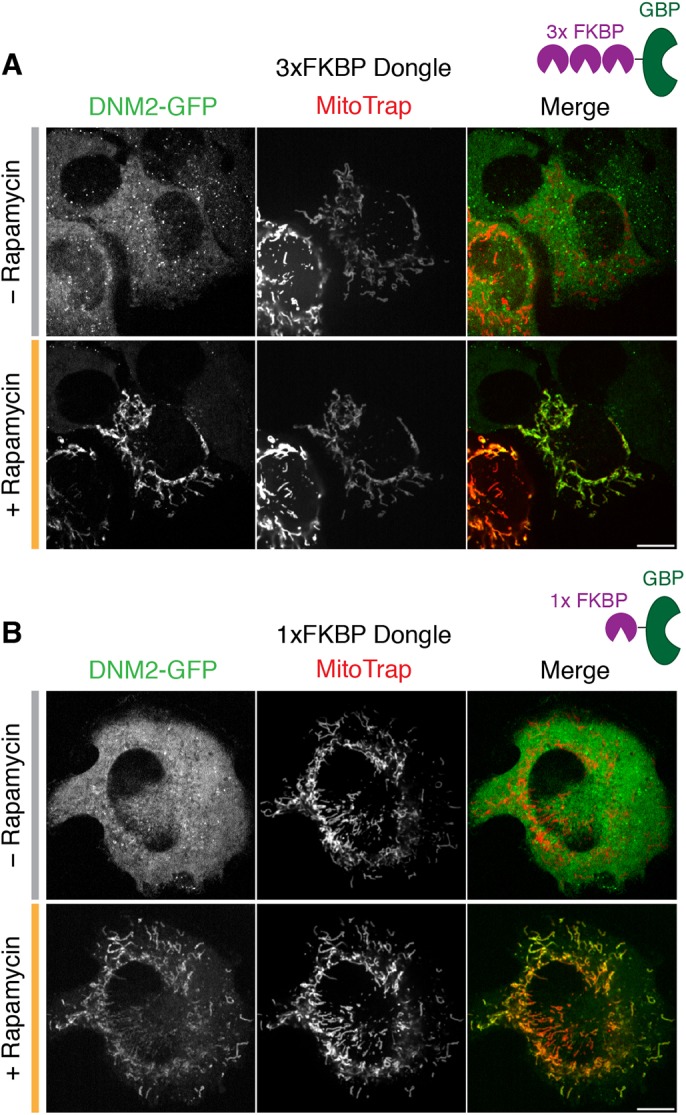
**Dongle-knocksideways efficiently reroutes dynamin-2–GFP to mitochondria.** Still confocal images from dongle-knocksideways experiments showing a cell before and after application of 200 nM rapamycin. SK-MEL-2 hDNM2^EN-all^ cells expressing GFP-tagged dynamin-2 (DNM2–GFP), MitoTrap and either (A) 3×FKBP dongle or (B) 1×FKBP dongle. Scale bars: 10 µm.

We next wanted to use the dongle-knocksideways method to switch off endocytosis on demand. A direct approach would be to inactivate the large GTPase dynamin, which is essential for vesicle scission during endocytosis (Antonny et al., 2016). We therefore tested dongle-knocksideways in SK-MEL-2 hDNM2^EN-all^ cells, where both alleles of dynamin-2 are tagged with GFP (Doyon et al., 2011). Confocal imaging revealed rapid and efficient rerouting of dynamin-2–GFP (DNM2–GFP) to mitochondria using 200 nM rapamycin in cells co-expressing the dongle and MitoTrap (Fig. 3A; Movie 1).

The dongle used for knocksideways has three FKBP domains in tandem, attached to GBPen (3×FKBP dongle). For reasons that will become clear below, we also generated a dongle with a single FKBP domain (1×FKBP dongle). Using this construct for dongle-knocksideways of DNM2–GFP in SK-MEL-2 hDNM2^EN-all^ cells was similar to experiments that used 3×FKBP dongle (Fig. 3B). Therefore, dynamin-2–GFP can be rerouted efficiently to mitochondria using dongle-knocksideways.

### Inhibition of clathrin-mediated endocytosis using dongles

**Fig. 4.**
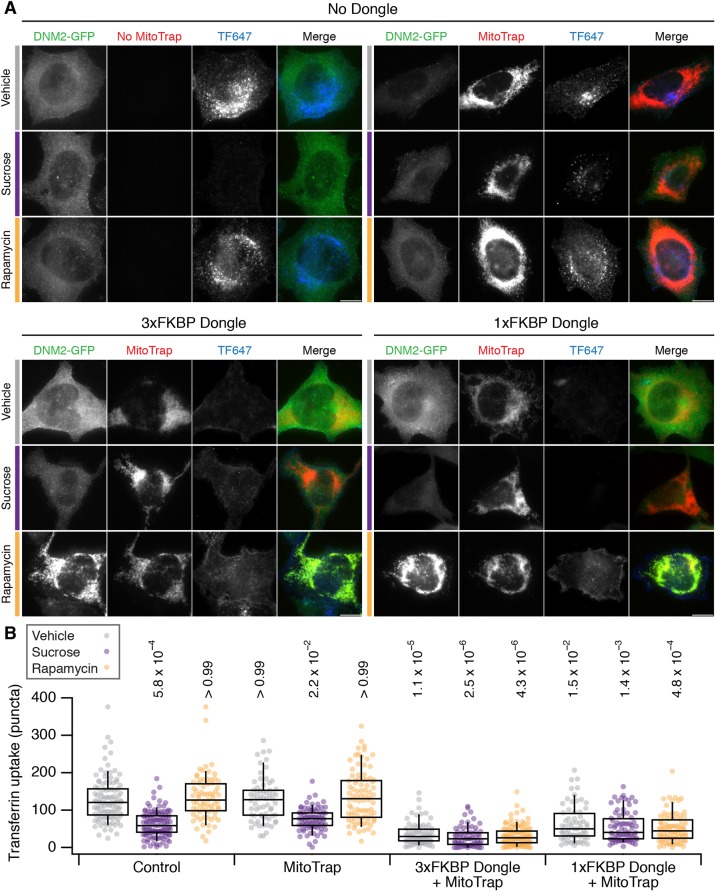
**Effect of dongle expression on transferrin uptake in SK-MEL-2 hDNM2^EN-all^ cells.** (A) Micrographs of SK-MEL-2 hDNM2^EN-all^ cells treated with vehicle (gray bar), sucrose (purple bar) or 200 nM rapamycin (orange bar). Cells were untransfected (No Dongle, No Mitotrap), or expressed MitoTrap alone, or MitoTrap with 3×FKBP dongle or 1×FKBP dongle. DNM2–GFP (green), MitoTrap (red) and transferrin–Alexa Fluor 647 (TF647, blue) are displayed using the same minimum and maximum value per channel for all images in the figure. Scale bars: 10 µm. (B) Box plot to show quantification of transferrin uptake. Expression and treatments are as indicated and colored as in A. Dots represent individual cells from multiple experiments. Box represents the interquartile range (IQR), the line the median and the whiskers the 9th and 91st percentile. *n*_cell_=64–114, *n*_exp_=7. Two-way ANOVA on experimental means, within subject; Factor A=expression, *DF*=2, *F*=12.44, *F*_c_=8.77, *P*<0.001; Factor B=treatment, *DF*=3, *F*=35.02, *F*_c_=7.05, *P*<0.001; A×B, *DF*=6, *F*=3.17, *F*_c_=5.12, *P*<0.001. *P*-values from Dunnett's post hoc test are shown above each plot (Control vehicle as the control group).

Does dongle-knocksideways of dynamin-2–GFP cause an inhibition of clathrin-mediated endocytosis (CME)? To answer this question we analyzed the cellular uptake of fluorescently labelled transferrin, an established assay for CME. We first verified that chronic inhibition of CME could be achieved by expression of DongleTrap (Fig. S1). Then we tested the effect of dongle-knocksideways (rapamycin versus vehicle) and compared this to inhibition of endocytosis (sucrose) using hypertonic media (Hansen et al., 1993). To our surprise, we found that expression of the dongle was sufficient to inhibit CME in SK-MEL-2 hDNM2^EN-all^ cells (Fig. 4). The amount of transferrin uptake in cells expressing MitoTrap together with 3×FKBP dongle was significantly reduced compared to untransfected cells or those expressing MitoTrap alone (Fig. 4). This unintended inhibition of CME was similar to treatment with hypertonic media, a classical method to inhibit endocytosis. We wondered whether the size of 3×FKBP dongle caused this inhibition and so we generated a 1×FKBP dongle, which was approximately half the size (3×FKBP dongle, 49.8 kDa; 1×FKBP dongle, 25.9 kDa), and verified that the 1×FKBP dongle was fully functional for rerouting experiments (Fig. 3B). Again, this dongle caused inhibition of CME by expression in SK-MEL-2 hDNM2^EN-all^ cells, similar to that seen for 3×FKBP dongle (Fig. 4). Similar results were seen using SK-MEL-2 hCLTA^EN^/hDNM2^EN^ cells, which indicated that this effect was not specific to the hDNM2^EN-all^ cell line used (Fig. S2). Note that with either dongle and either cell line, no further inhibition of CME was observed by sucrose treatment nor by rapamycin addition causing dongle-knocksideways. These observations mean that the dongle method cannot be used in this way to inhibit endocytosis on demand, since the active state is inhibited unintentionally.

**Fig. 5.**
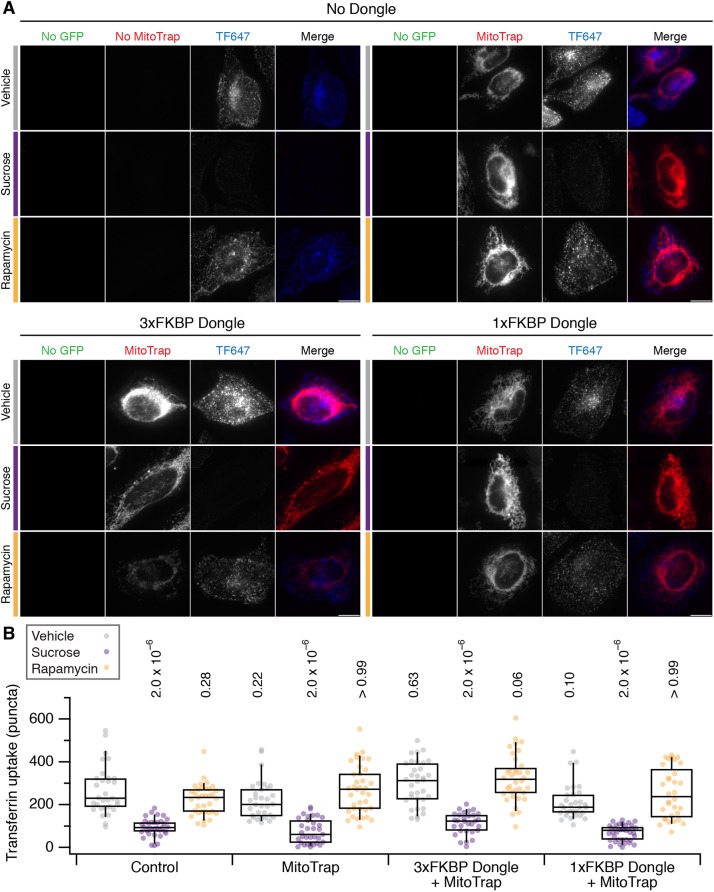
**Effect of dongle expression on transferrin uptake in HeLa cells.** (A) Micrographs of HeLa cells treated with vehicle (gray bar), sucrose (purple bar) or 200 nM rapamycin (orange bar). Cells were untransfected (No Dongle, No Mitotrap), or expressed MitoTrap alone, or MitoTrap with 3×FKBP dongle or 1×FKBP dongle. No GFP (green), MitoTrap (red) and transferrin–Alexa Fluor 647 (TF647, blue) are displayed using the same minimum and maximum value per channel for all images in the figure. Scale bars: 10 µm. (B) Box plot to show quantification of transferrin uptake. Expression and treatments are as indicated and colored as in A. Dots represent individual cells from a single experiment. Box represents the IQR, the line the median and the whiskers the 9th and 91st percentile. One-way ANOVA *P*-values from Dunnett's post hoc test are shown above each plot (Control vehicle as the control group).

Our results suggested that the unintentional inhibition of CME is caused by dongles binding to dynamin-2–GFP and inhibiting its function. An alternative hypothesis is that the dongles inhibit CME via some unknown mechanism and the effect in cells with dynamin-2–GFP was coincidental. To test whether dongles inhibited CME directly, we measured transferrin uptake in HeLa cells with no dynamin-2–GFP, which expressed either the 3×FKBP or 1×FKBP dongles with MitoTrap (Fig. 5). We found that transferrin uptake in these cells was similar to that in cells expressing MitoTrap alone or to untransfected controls (Fig. 5). CME in cells expressing either dongle could be inhibited by sucrose and not by rapamycin treatment, which is to be expected if there is no direct inhibition of CME caused by the dongle. These experiments ruled out an inhibitory effect of dongles on CME, and implicate the inhibition seen in cells expressing dynamin-2–GFP as being due to binding of dynamin-2–GFP with the nanobody.

### Unintended perturbation of GFP–TPD54 function with dongles

**Fig. 6.**
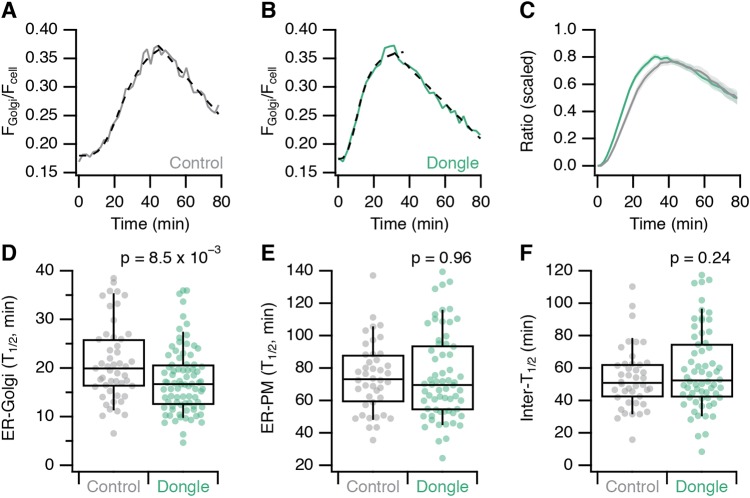
**Effect of dongle expression on anterograde in GFP–TPD54 knock-in HeLa cells.** (A,B) Example trace of the E-cadherin fluorescence ratio following RUSH recorded from (A) a control (MitoTrap-expressing, gray) or (B) a dongle (3×FKBP dongle-expressing, green) GFP–TPD54 knock-in HeLa cell. Traces fitted with a logistic function and a line. (C) Scaled fraction of total E-cadherin fluorescence at the Golgi as a function of time, in control (gray) or dongle-expressing (green) cells. Line and shaded area represent mean±s.e.m. (D–F) Box plots showing the half-times of E-cadherin transport from (D) ER-to-Golgi and (E) ER-to-PM in control and dongle-expressing GFP–TPD54 knock-in cells. (F) The difference in half-times represents intra-Golgi transport. Box represents the IQR, the line the median and the whiskers the 9th and 91st percentile. *P*-values from Student's *t*-test with Welch's correction. *n*_cell_=49–82, *n*_exp_=3.

The dongle-mediated inhibition of dynamin-2–GFP function might be particular to dynamin-2. We therefore tested whether the function of GFP–TPD54 was also affected by dongle expression. TPD54 is involved in anterograde traffic and its depletion results in delayed transit of E-cadherin in a retention using selective hooks (RUSH) assay (Larocque et al., 2019; Boncompain et al., 2012). In GFP–TPD54 knock-in HeLa cells, we analysed the export of SBP–mRuby2–E-cadherin from the ER to Golgi and on to the plasma membrane using RUSH (Fig. 6). In cells that express dark MitoTrap and 3×FKBP dongle, export from the ER to Golgi was significantly faster compared to cells expressing dark MitoTrap alone (control) (Fig. 6A–D). The kinetics of transport from the Golgi to the plasma membrane were unaffected. These experiments indicate that unintended perturbation of GFP-tagged protein function is not particular to dynamin-2 but may be a general consequence of dongle expression.

## DISCUSSION

In this paper, we described the development of dongles to extend the functionality of GFP for knocksideways experiments. We found that these molecular tools were effective at binding GFP and permitting the rerouting of the target protein to mitochondria. However, we discovered an unintended side effect: dongles can perturb the function of the GFP-tagged protein under study.

The use of GFP-binding proteins as an intracellular tool to manipulate protein function is becoming widespread (Prole and Taylor, 2019; Daniel et al., 2018; Ariotti et al., 2015). The appeal of the method is that proteins tagged with GFP at their endogenous loci can be adapted using dongles to enable inactivation, relocalization, recoloring or other function. This means that existing cell lines or organisms can be ‘retrofitted’ for additional functionality using such tools. Indeed, our initial experiments using dongles were very encouraging; the dongles expressed well, we detected no obvious perturbation of subcellular distribution of the target proteins we examined, and they permitted dongle-knocksideways which, in the case of TPD54, was very similar to knocksideways (rerouting the same protein with a fused GFP–FKBP tag). However, we found that when the dongles were expressed in cells with both copies of dynamin-2 tagged with GFP, endocytosis was inhibited. This unintended effect seemed to be due to direct inhibition of dynamin function following binding of dynamin-2–GFP by the nanobody (GBPen) portion of the dongle. Dynamins may be uniquely sensitive because they self-oligomerize, and we know that their action can be readily inhibited by simple expression of a GTPase deficient isoform (Damke et al., 1994). However, this problem was not unique to dynamin-2 since we also saw that GFP–TPD54 function in anterograde traffic was perturbed by dongle expression.

We were fortunate to test this method on dynamin-2, which has a clear functional readout as it controls the terminal step in clathrin-mediated endocytosis (Antonny et al., 2016). Other proteins do not have such unambiguous readouts, or their function can only be measured indirectly, if at all. This would mean that if dongles were used with these proteins, perturbed function would not be revealed and potentially misleading conclusions drawn. This problem is compounded because dongles would be most useful when applied to proteins whose function is uncertain, so the perturbation of protein function caused by these tools may be hidden from the investigator. Our advice is that the same caution and functional tests should be applied when using GFP nanobodies in cells as when generating GFP-tagged proteins themselves (Snapp, 2005).

The mechanism of inhibition of dynamin-2–GFP function by dongles is unclear. The simplest explanation is that extending the GFP tag using a GFP nanobody plus additional domains results in a modification that is simply too large for dynamin to function normally; whether this is because of reduced dynamics, blocked interactions or some other mechanism. We saw similar unintended inhibition when the size of the dongle was reduced by half (from three FKBP domains to one), suggesting that the binding of the nanobody itself is inhibitory rather than there being a size limit to the tag that dynamin-2 can tolerate. It is considered that most proteins can tolerate the addition of GFP or GFP–FKBP tag, but it is perhaps underappreciated that at some level, tags will always interfere with protein function. The fact that we report perturbed protein function using dongles is perhaps not surprising.

Unintended inhibition affects experiments where the protein of interest needs to be functional (active) prior to inactivation. For other applications of dongles, inhibition may not be such a concern. First, in constitutive mislocalization experiments, where the goal is to chronically inactivate protein function by changing its cellular localization, dongles remain an important tool. We demonstrated here the use of DongleTrap to inactivate dynamin-2–GFP. Second, it is unclear whether labeling strategies based on dongles are compromised by inhibition (Ariotti et al., 2015). We saw no evidence of gross changes in subcellular localization of the two proteins we tested; however, it remains questionable whether imaging a protein of interest in its inhibited state is representative of its normal distribution. Third, in cases where investigators simply want to put a functional domain to a new location using a GFP-tagged anchor protein, such as calcium sensors at the endoplasmic reticulum (Prole and Taylor, 2019), inhibition of the anchor may not be a concern. Fourth, our finding of nanobody-mediated inactivation of protein function may even be useful as a general purpose method for perturbing protein function in gene-edited cell lines.

What is the best strategy to extend the functionality of tags introduced with knock-in technology? First, it may be possible to reduce the inhibitory effect of dongles by mutating the GBP moiety or using different domain configurations and/or by changing the linker regions. Second, alternative GFP-binding proteins such as those based on a designed ankyrin repeat protein (DARPin) scaffold may be functionalized and used as dongles (Brauchle et al., 2014). It is possible that these reagents do not have the same inhibitory effects. Third, using split-GFP technology, proteins of interest could be tagged with GFP11 and then the fluorescence complemented with a GFP1-10 protein (Kamiyama et al., 2016), where GFP1-10 is fused to other domains to extend the functionality. A further advantage of this third method is that the fluorescence of the tagged protein can also be altered during the complementation (Kamiyama et al., 2016). However, a weakness is that this method would not take advantage of existing GFP-tagged collections, and would require new knock-ins to be generated in most cases. Finally, while GBPen is the most widely used GFP nanobody, it is possible that dongles that incorporate alternative GFP nanobodies may cause less perturbation to target protein function.

## MATERIALS AND METHODS

### Molecular biology

Construction of plasmids to express GFP–TPD54 and GFP–FKBP–TPD54 and mCherry-MitoTrap (pMito-mCherry-FRB) was described previously (Cheeseman et al., 2013; Larocque et al., 2019). The nanobody cDNA used in this paper, described as GBPen (GFP-binding protein enhancer), was synthesized from published sequences (Kubala et al., 2010; Kirchhofer et al., 2010). To make pMito-mCherry-FRB-IRES-FKBP(III)-GBPen, a bicistronic vector to co-express mCherry-MitoTrap and 3×FKBP–GBPen via an internal ribosome entry site (IRES), a custom insert was made using gene synthesis (GenScript) and inserted into pEGFP-C1 in place of GFP at AgeI and EcoRI. A dark version of pMito-mCherry-FRB-IRES-FKBP(III)-GBPen was also made by site-directed mutagenesis in mCherry to include the K70N mutation. To express DongleTrap, pMito-GBPen was made by amplifying GBPen from a plasmid containing FKBP(III)-GBPen (forward: 5′-cttaggatccggcaCAGGTGCAGCTG-3′, reverse: 5′-ggcctctagaTCAATGGTGATGGTG-3′) cloning into demethylated pMito-mCherry-FRB using BamHI and XbaI. To make pMito-mCherry-FRB-IRES-FKBP(I)-GBPen, the region of IRES including the HindIII cut site and 1×FKBP was amplified using PCR from pMito-mCherry-FRB-IRES-FKBP(III)-GBPen with addition of a BglII site at the end of the amplified fragment (forward: 5′-GTTCCTCTGGAAGCTTCTTGAAG-3′, reverse: 5′-gcgagatctTTCCAGTTTTAGAAGCTCCACATC-3′). The product was cut with HindIII and BglII. The same vector was cut with HindIII and BglII, resulting in a vector lacking all three FKBP tags. The cut PCR product was ligated back into the cut vector. pMito-mCherry-FRB-IRES-FKBP(I)-GBPen, pMito-mCherry-FRB-IRES-FKBP(III)-GBPen and pMito-GBPen were deposited to Addgene as #128267, #128268, #128269, respectively. To make Str-KDEL-SBP-mRuby2-E-Cadherin, the FastCloning technique was used (Li et al., 2011). Briefly, EGFP was removed from the original RUSH construct Str-KDEL-SBP-EGFP-E-Cadherin (Boncompain et al., 2012) by amplifying the plasmid from either side of EGFP with the following primers: forward: 5′-GGACGAGCTGTACAAGGGccggCCAgactgggtc-3′ and reverse: CGCCCTTAGACACCATACCtgcaggTGGTTCACGTTG-3′. mRuby2 was amplified using mRuby2-N1 (Addgene, #54614) with the following primers: forward: 5′-GAACCAcctgcaGGTATGGTGTCTAAGGGCGAAGAG-3′ and reverse: 5′-ccagtcTGGccggCCCTTGTACAGCTCGTCCATCCC-3′. Both amplicons have overlapping sequences. The insert and the vector were then digested with DpnI and transformed in DH5α cells.

Plasmids to express fluorescent proteins were either available from previous work: pDsRed-N1, pEGFP-N1, pECFP-N1, pEYFP-N1, pmRFP-N1, pmCherry-N1, pTagBFP2; from Addgene: pmScarlet-C1 (#85042), pTagRFP657-N1 (#44275), psfGFP-N1 (#54737), pEBFP2-N1 (#54595), mAzurite-N1 (#54617), mCerulean3-N1 (#54730), mTurquoise2-N1 (#60561), mVenus-N1 (#27793), mRuby2-N1 (#54614), mNeptune2-N1 (#54837), mOrange2-N1 (#54499), mCitrine2-N1 (#54594), mEmerald-N1 (#53976), pcDNA3-Clover (#40259), mAzamiGreen-N1 (#54798), mMaroon-N1 (#54554), mKO2-N1 (#54625); or from Allele Biotech: pmNeonGreen-N1 (ABP-FP-MNEONSA).

### Cell biology

HeLa cells (HPA/ECACC #93021013) or GFP–TPD54 knock-in HeLa cells (Larocque et al., 2019) were cultured in DMEM+GlutaMAX (Thermo Fisher) supplemented with 10% fetal bovine serum, and 100 U ml^−1^ penicillin/streptomycin. SK-MEL-2 hDNM2^EN-all^ or hDNM2^EN-all^/CLTA^EN^ cells (a kind gift from David Drubin, Department of Molecular and Cellular Biology, University of California Berkeley, CA) were cultured in Dulbecco's modified Eagle's medium or nutrient mixture F-12 Ham (Sigma-Aldrich) supplemented with 10% fetal bovine serum, 1% L-glutamine, 3.5% sodium bicarbonate and 100 U ml^−1^ penicillin/streptomycin. All cells were kept at 37°C and 5% CO_2_. HeLa cells were transfected with 1.2 µg DNA (total) per 3 µl GeneJuice (Merck Millipore) according to the manufacturer's instructions. SK-MEL-2 cells were transfected with 4.8 µg DNA (total) per 850,000 cells using Neon Transfection System (Thermo Fisher) with three pulses of 1500 V, 10 ms. Cells were analyzed 2 days post-transfection.

Transferrin uptake experiments were as described previously (Clarke and Royle, 2018). Briefly, cells were serum-starved for 30 min. For knocksideways, they were exposed to 200 nM rapamycin (Alfa Aesar) or 0.1% ethanol (vehicle) for the last 10 min of starvation, and then incubated with 100 µg/ml Alexa Fluor 647-conjugated transferrin (Invitrogen) for 10 min. Hypertonic sucrose media (0.45 M) was used to inhibit transferrin uptake. All incubations were in serum-free media at 37°C with 5% CO_2_ in a humidified incubator. Cells were then fixed in 3% PFA/4% sucrose in PBS and mounted on slides using Mowiol.

### Microscopy

For live-cell imaging of rerouting experiments, cells were grown in 4-well glass-bottom 3.5 cm dishes (Greiner Bio-One) and media exchanged for Leibovitz L-15 CO_2_-independent medium. Rerouting was triggered by addition of 200 nM rapamycin in L-15 media.

For the RUSH assay, GFP–TPD54 knock-in HeLa cells were transfected with Str-KDEL-SBP-mRuby2-E-Cadherin and either dark MitoTrap (Wood et al., 2017) alone or a plasmid that expressed 3×FKBP dongle and dark MitoTrap coupled by an IRES. mRuby2–E-cadherin was released from the ER by adding a final concentration of 40 µM D-Biotin (Sigma-Aldrich) in L-15 medium. Images were captured at a time interval of 2 min.

All cells were imaged at 37°C on a spinning disc confocal system (Ultraview Vox, PerkinElmer) with a 100×1.4 NA oil-immersion objective. Images were captured using an ORCA-R2 digital CCD camera (Hamamatsu) following excitation with 488 nm and 561 nm lasers.

Imaging of fixed cells was done on a Nikon Ti-U epiflorescence microscope with 100× oil-immersion objective, CoolSnap MYO camera (Photometrics) using NIS-Elements software (Nikon).

### Data analysis

Analysis of transferrin uptake was done as described previously (Wood et al., 2017). Briefly, single cells were outlined manually in Fiji. Vesicular structures were isolated by applying a manual threshold to images in the transferrin channel. Positive structures were counted using ‘Analyze particles’, with limits of 0.03–0.8 µm and circularity of 0.3–1.0. All analysis was done with the experimenter blind to the conditions of the experiment.

Analysis of RUSH experiments was done as previously described (Larocque et al., 2019), using custom-written code (https://doi.org/10.5281/zenodo.3366083).

Figures were made with Fiji or Igor Pro 8 (WaveMetrics), and assembled using Adobe Illustrator. Null hypothesis statistical tests were done as described in the figure legends.

## Supplementary Material

Supplementary information
