# Supplementary material for: Unintended perturbation of protein function using GFP nanobodies in human cells
Source: J Cell Sci. 2019 Nov 6;132(21):jcs234955. doi: 10.1242/jcs.234955 (PMC6857592; doi:10.1242/jcs.234955)
Supplement: Supplementary information [file joces-132-234955-s1.pdf]

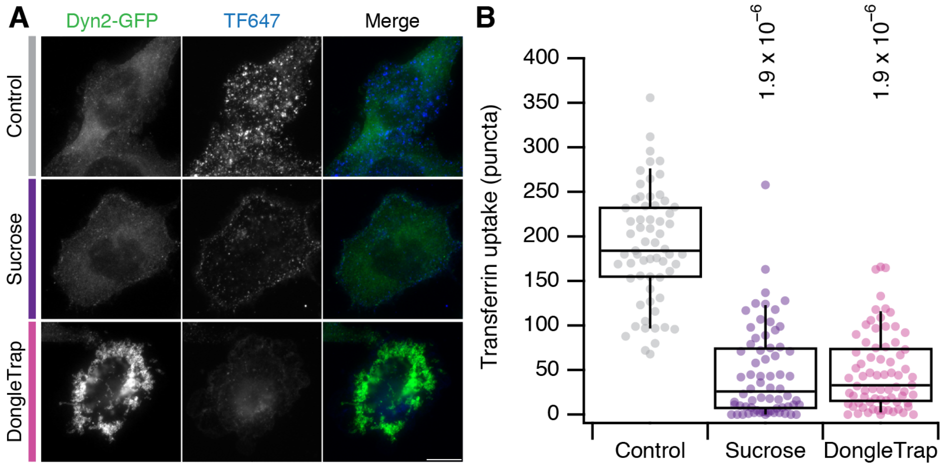

Figure S1. DongleTrap inhibits clathrin-mediated endocytosis in SK-MEL-2 hDNM2$^{\text{EN-all}}$ cells

A, Micrographs of SK-MEL-2 hDNM2$^{\text{EN-all}}$ cells. Untransfected cells, control (gray) or sucrose-treated (purple), or cells expressing DongleTrap (pink) are shown. DNM2-GFP (green)and transferrin-Alexa647 (blue) are displayed using the same minimum and maximum value per channel for all images in the figure. Scale bar, 10 µm.

B, Box plot to show quantification of transferrin uptake. Expression or treatments are as indicated and colored as in A. Dots represent individual cells from multiple experiments. Box represents the IQR, the line the median and the whiskers the 9th and 91st percentile. $n_{\text{cell}} = 49 - 68$, $n_{\text{exp}} = 3$. P-values from Dunnett's *post hoc* test are shown (Compared to Control).

Journal of Cell Science • Supplementary information

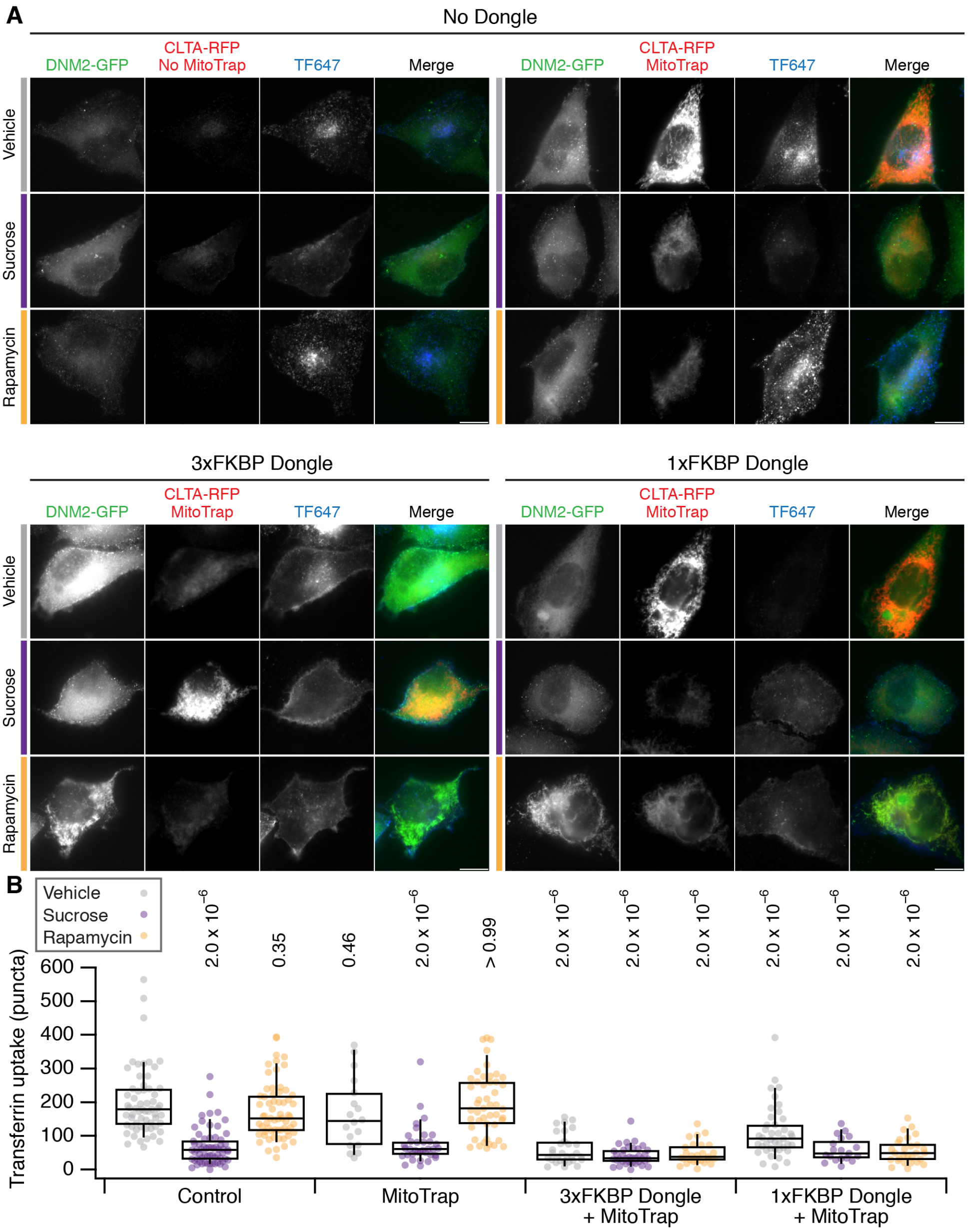

Figure S2. Effect of dongle expression on transferrin uptake in SK-MEL-2 hCLTA^{EN}/hDNM2^{EN} cells

A, Micrographs of SK-MEL-2 hCLTA^{EN}/hDNM2^{EN} cells treated with vehicle (gray), sucrose (purple) or 200 nM rapamycin (orange). Cells were untransfected (No Dongle, No Mitotrap), or expressed MitoTrap alone, or MitoTrap with 3xFKBP Dongle or 1xFKBP Dongle. DNM2-GFP (green), CLTA-RFP + MitoTrap (red) and transferrin-Alexa647 (blue) are displayed using the same minimum and maximum value per channel for all images in the figure. Note that MitoTrap and CLTA-RFP appear in the same channel. Scale bars, 10 µm.

B, Box plot to show quantification of transferrin uptake. Expression and treatments are as indicated and colored as in A. Dots represent individual cells from multiple experiments. Box represents the IQR, the line the median and the whiskers the 9th and 91st percentile. P-values from Dunnett's *post hoc* test are shown (Control-Vehicle as the control group). $n_{\text{cell}} = 19 - 64$, $n_{\text{exp}} = 3$.

Journal of Cell Science • Supplementary information

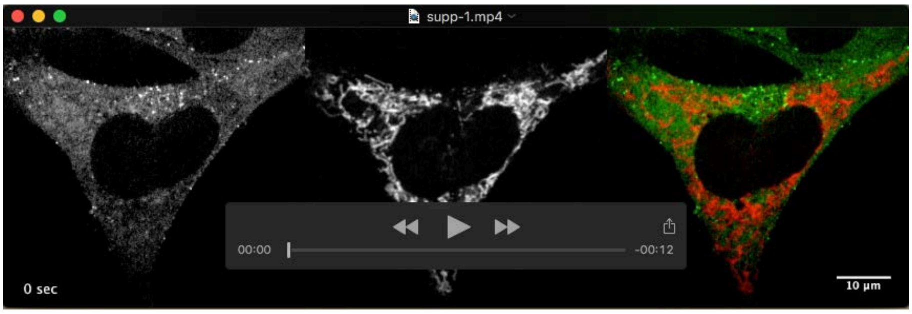

Movie 1. Knocksideways of DNM2-GFP using 3xFKBP Dongle.
Live cell confocal microscopy of dongle-knocksideways, 200 nM rapamycin is added at 10 s. Dynamin-2-GFP (left, green) and MitoTrap (middle, red) are shown together with a merge (right).
Time, seconds. Scale bar, 10 µm.
